# Supplementary material for: Activation Patterns of Functional Brain Network in Response to Action Observation-Induced and Non-Induced Motor Imagery of Swallowing: A Pilot Study
Source: Brain Sci. 2022 Oct 21;12(10):1420. doi: 10.3390/brainsci12101420 (PMC9599111; doi:10.3390/brainsci12101420)
Supplement: Supplementary file 1 [file brainsci-12-01420-s001.zip › brainsci-1937329-supplementary.pdf]

Supplementary table 1. The abbreviation of brain regions corresponds to the full name in the text.

Abbreviation	Full name	Abbreviation	Full name
AnguGyr_Mid_L	angular gyrus_middle L	AnguGyr_Pst_R	angular gyrus_posterior R
AntObtFrtGyr_L	anterior orbito-frontal gyrus L	AntObtFrtGyr_R	anterior orbito-frontal gyrus R
GyrRectus_L	gyrus rectus L	GyrRectus_R	gyrus rectus R
Insula_Ant_L	insula_anterior L	Insula_Pst_R	insula_posterior R
Insula_Pst_L	insula_posterior L	MidFrtGyr_Pst_R	middle frontal gyrus_posterior R
ParsTriagu_Pst_L	pars triangularis_posterior L	MidObtFrtGyr_R	middle orbito-frontal gyrus R
PstObtFrtGyr_L	posterior orbito-frontal gyrus L	PstObtFrtGyr_R	posterior orbito-frontal gyrus R
TsvFrtGyr_Lat_L	transverse frontal gyrus_lateral L	TsvFrtGyr_Msl_R	transverse frontal gyrus_mesial R
LatObtFrtGyr_Ant_L	lateral orbitofrontal gyrus_anterior L	LatObtFrtGyr_Pst_R	lateral orbitofrontal gyrus_posterior R
SupPariGyr_Pst_L	superior parietal gyrus_posterior L	ParaCentLob_R	paracentral lobule R
ParaCentLob_L	paracentral lobule L	PostCentGyr_Sup_R	postcentral gyrus_superior R
PostCentGyr_Sup_L	postcentral gyrus_superior L	PreCentGyr_Sup_R	precentral gyrus_superior R
PreCentGyr_Inf_L	precentral gyrus_inferior L	Cune_Pst_R	cuneus_posterior R
PreCentGyr_Sup_L	precentral gyrus_superior L	InfOcciGyr_Ant_R	inferior occipital gyrus_anterior R
MidFrtGyr_Ant_L	middle frontal gyrus_anterior L	LingualGyr_Pst_R	lingual gyrus_posterior R
ParsOrbitalis_L	pars orbitalis L	MidOcciGyr_Pst_R	middle occipital gyrus_posterior R
SprmarGyr_Ant_L	supramarginal gyrus_anterior L	SupOcciGyr_Sup_R	superior occipital gyrus_superior R
LingualGyr_Pst_L	lingual gyrus_posterior L	SupFrtGyr_Ant_R	superior frontal gyrus_anterior R
CingGyr_Pst_L	cingulate gyrus_posterior L	SupFrtGyr_Pst_R	superior frontal gyrus_posterior R
PreCune_Inf_L	precuneus_inferior L	MidTepGyr_DsoPst_R	middle temporal gyrus_dorsoposterior R
PreCune_Sup_L	precuneus_superior L	MidTepGyr_VenPst_R	middle temporal gyrus_ventroposterior R
SubcallosalGyr_L	subcallosal gyrus L	ParaHippoGyr_R	parahippocampal gyrus R
FusiGyr_Ant_L	fusiform gyrus_anterior L	TsvTepGyr_R	transverse temporal gyrus R
FusiGyr_Pst_L	fusiform gyrus_posterior L	AnguGyr_Ant_R	angular gyrus_anterior R
InfTepGyr_Mid_L	inferior temporal gyrus_middle L	ParsTriagu_Mid_R	pars triangularis_middle R
InfTepGyr_Pst_L	inferior temporal gyrus_posterior L	TsvFrtGyr_Lat_R	transverse frontal gyrus_lateral R
MidTepGyr_DsoPst_L	middle temporal gyrus_dorsoposterior L	LatObtFrtGyr_Ant_R	lateral orbitofrontal gyrus_anterior R
MidTepGyr_Mid_L	middle temporal gyrus_middle L	SupPariGyr_Pst_R	superior parietal gyrus_posterior R
MidTepGyr_VenPst_L	middle temporal gyrus_ventroposterior L	ParsOpcu_Inf_R	pars opercularis_inferior R
TepPole_L	temporal pole L	PreCune_Inf_R	precuneus_inferior R
Cune_Ant_L	cuneus_anterior L	FusiGyr_Ant_R	fusiform gyrus_anterior R
MidOcciGyr_DsoAnt_L	middle occipital gyrus_dorsoanterior L	MidTepGyr_Ant_R	middle temporal gyrus_anterior R
MidOcciGyr_VenAnt_L	middle occipital gyrus_ventroanterior L	SupTepGyr_Ant_R	superior temporal gyrus_anterior R
SupFrtGyr_Ant_L	superior frontal gyrus_anterior L	SupTepGyr_Mid_R	superior temporal gyrus_middle R
SupFrtGyr_Pst_L	superior frontal gyrus_posterior L	SupTepGyr_Pst_R	superior temporal gyrus_posterior R
SupTepGyr_Mid_L	superior temporal gyrus_middle L	SprmarGyr_Pst_R	supramarginal gyrus_posterior R
AnguGyr_Ant_L	angular gyrus_anterior L	SupPariGyr_Ant_R	superior parietal gyrus_anterior R
InfOcciGyr_Ant_L	inferior occipital gyrus_anterior L	SprmarGyr_Ant_R	supramarginal gyrus_anterior R
InfOcciGyr_VenPst_L	inferior occipital gyrus_ventroposterior L	ParsTriagu_Pst_R	pars triangularis_posterior R
TsvTepGyr_L	transverse temporal gyrus L	ParsOrbitalis_R	pars orbitalis R

ParsOpcu_Sup_L	pars opercularis_superior L
InfOcciGyr_DsoPst_L	inferior occipital gyrus_dorsoposterior L
SupTepGyr_Ant_L	superior temporal gyrus_anterior L
MidFrtGyr_Pst_L	middle frontal gyrus_posterior L
SprmarGyr_Pst_L	supramarginal gyrus_posterior L
